# Support for affirmative actions to increase inclusivity of intersex* persons at an Austrian medical university

**DOI:** 10.1186/s12909-023-04830-z

**Published:** 2023-11-03

**Authors:** Judith Walser, Margarethe Hochleitner, Nikola Komlenac

**Affiliations:** 1 Gender Medicine Unit, Medical University of Innsbruck, Innsbruck, 6020 Austria; 2 Faculty of Business and Management, University of Innsbruck, Innsbruck, Austria

**Keywords:** Intersex variation, Knowledge test, Affirmative actions, Medical students, Medical university staff, Austria

## Abstract

**Background:**

Since the ruling handed down by the Austrian Constitutional Court in 2018 intersex variation has been recognized under Austrian law as a “third sex”. In order to ensure that people with intersex variation are not discriminated against based on their group membership affirmative actions (i.e., proactive practices to avert discrimination) can be implemented. The current study explored whether students and employees at an Austrian medical university know about intersex variation. Furthermore, the study investigated what affirmative actions are practiced at the medical university to be inclusive for intersex persons and students’ and employees’ support for such affirmative actions.

**Methods:**

All students and employees of a medical university in Austria were invited by e-mail to participate at the current study that included a self-constructed knowledge test on intersex variation with ten true-false questions. On five-point Likert scales participants reported for each of twelve listed affirmative actions whether they had seen a certain affirmative action at their university and how important they thought the implementation of an affirmative action was. Finally, participants’ gender, age, sexual orientation, highest level of education, and nationality was assessed. A cluster analysis was performed to determine groups of people with different degrees of support for affirmative actions for intersex persons.

**Results:**

220 students (62% cisgender women, 38% cisgender men) and 200 employees (72% cisgender women, 28% cisgender men) participated. Participants responded correctly to three out of ten knowledge test questions. The cluster analysis revealed that participants could be clustered as heterosexual cisgender women (Cluster 1; 55%), heterosexual cisgender men (Cluster 2; 30%), or sexual minority cisgender women and men (Cluster 3; 15%). Sexual minority persons knew more about intersex variation than did heterosexual participants. On average, affirmative actions for the inclusivity of intersex people have not been encountered (*M* = 1.5, *SD* = 0.4) at the studied university. Participants, especially those in Cluster 3, believed that the listed actions are moderately important.

**Conclusions:**

At the medical university many actions should be taken to increase inclusivity for intersex people. Increasing the knowledge of university staff and students concerning intersex might help increase their support for such actions.

**Supplementary Information:**

The online version contains supplementary material available at 10.1186/s12909-023-04830-z.

## Background

Intersex persons are individuals with a congenital variation in the development of sex, such that their chromosomal, gonadal, and/or anatomical sex (primary and secondary sex characteristics) development does not allow them to be clearly classified as either male or female [1–3]. Those variations can entail physical differences (without physiological differences) between intersex people and people who are classified as male or female. In the past intersex variation has been pathologized (i.e., has been labelled as wrong, abnormal, or disordered) [4], which often lead to genital surgical procedures to “correct” intersex persons’ genitalia during infancy so that they conform standards of how genitalia are expected to be [5–8]. Such genital surgical procedures in infancy are not only unnecessary surgical interventions, but can be understood as human rights violation and have profound negative medical and psycho-social consequences for the intersex individual [5–8].

Current guidelines on the medical care of intersex people [9] focus on syndromes that can be associated with certain intersex variations [10, 11]. An European multicenter study among 1,040 adults with intersex variation revealed that 84.3% of intersex persons reported at least one medical problem associated with their intersex variation [12], such as endocrine disorders, heightened gonadal tumor risk, risk of urological complications, reduced growth and bone development, or problems with infertility. For those syndromes intersex persons profit from medical care provided by a multidisciplinary team [9, 11, 13]. As a result, most intersex people (91.4%) people report fair to very good general health [12, 14].

Since 2018, Austrian law has officially recognized intersex variation as a “third sex” and intersex people are no longer forced to fit into the sex categories female or male, but can report their sex as “inter”/”third sex” in official documents [15, 16]. Official and legal recognition is one significant step forward in reducing discrimination of intersex persons and in de-pathologizing intersex variation. Nevertheless, intersex people experience often forms of minority stress [17, 18] when being discriminated, fearing or facing stigma, being bullied, or being rejected or excluded based on not meeting cis-heteronormative expectations (i.e., the expectation that there are only two sexes that are categorically different, that gender is determined by biological characteristics, and that people are always sexually attracted to people of “the other” gender [19]) [7, 20]. Intersex persons living in Austria have reported experiencing such and similar forms of minority stress in many life situations [21]. Experiencing prolonged and frequent minority stress can lead to psychological and medical health problems that can seriously increase intersex persons’ mortality risk [22, 23]. Recently, it was emphasized that not only the experience of minority stress, but also reduced or absent social safety (i.e., having reliable social connections, a sense of social belongingness, experiencing social inclusion, or social recognition) of stigmatized individuals can be detrimental for those individuals’ health [24].

In order to increase the sense of social safety and to make sure that intersex people are not discriminated against, affirmative actions can be implemented in organizations [25]. Affirmative actions can be defined as the organization’s devotion of resources to proactively avert discrimination of stigmatized or minority groups in an organization. For universities some affirmative actions have been recommended to increase sexual- and gender-minority persons inclusivity [26]. Those affirmative actions include educational programs and discussions during meetings or lectures that inform about the disadvantages minority people experience in everyday life. Other affirmative actions can be written guidelines and policies against discrimination of minority people [27–29]. The change of university forms to include more than binary categories of sex and gender can be an affirmative action. E-mail correspondence can include preferred pronouns and can address students and employees by using gender-neutral language. Visibility and messages of inclusion can be increased by symbols that transport inclusivity of sexual- and gender-minority persons [25, 26, 30]. Gender-segregated spaces (including gender-segregated restrooms [27–29, 31]) that reinforce sex and gender binarism [32] can be changed to be inclusive to all people (i.e., implementation of all gender restrooms) [27–29, 31]. Overall, affirmative actions can address stigmatizing policies, non-inclusive language and forms, misgendering, gendered cultural norms, unequal access to restrooms, lack of visibility, or lack of knowledge about sexual and gender diversity [27, 28, 33].

Attitudes toward and support for affirmative actions can vary [26]. Past research has reported that women, as compared to men, are more likely to recognize the relevance of affirmative actions for intersex persons [34, 35]. Women have been found to more strongly wish for the implementation of affirmative actions for the benefit of marginalized groups at universities than do men [36]. Finally, past findings have also shown that sexual minority persons are more supportive for affirmative actions for intersex persons than are heterosexual persons [34–36].

## Aim of the current study

The current explorative study assessed what and how many of the recommended affirmative actions for universities [27, 28, 33] have been implemented at an Austrian medical university with a view to increasing the inclusivity of intersex persons. Additionally, students’ and employees’ perceptions of the importance of and their wish for such affirmative actions have been assessed while also taking students’ and employees’ heteronormative attitudes and beliefs as well as their knowledge about intersex variation into account. Finally, in the current study a cluster analysis was performed to analyze whether people differing in gender and sexual orientation differed in their support for affirmative actions for intersex persons.

## Methods

### Procedures

After receiving confirmation from the medical university’s Ethics Committee that under Austrian law the current study did not require formal approval by an ethics committee [37, 38], the study was hosted on *SoSci: der onlineFragebogen* (http://soscisurvey.de/). An invitation e-mail to participate in the study was sent to all students and all employees at the Austrian medical university. The online questionnaire was accessible from February 2021 to August 2021. Participation was voluntary, anonymous and not associated with any incentive or compensation for participation. Participants were able to access the questionnaire only after agreeing that their anonymous data was saved and used for research.

In total 880 persons entered the questionnaire. Of those, 454 participants were excluded from the analysis because they either discontinued the survey before reaching two attention-check items (“Please select the response ‘Agree’”) or because they did not respond correctly to those items [39]. The number of participants who had a nonbinary, transgender, or other gender identity (*n* = 4) as well as those who did not indicate their gender (*n* = 2) was too small for meaningful analysis. Therefore, only people who identified as cisgender women or cisgender men were included in the analysis.

### Measures

#### Sociodemographic information

Sociodemographic information was assessed with self-constructed questions about participants’ age, gender, sexual orientation [40], highest level of education, and nationality. Participants were also asked whether they were students or employees at the medical university. For each question, participants could choose from several response options and give a free text response. For the analysis only cisgender women and cisgender men were considered, and the variables sexual orientation (heterosexually vs. sexual minority), highest level of education (school or vocational training vs. university) and nationality (Austrian vs. other) were dichotomized.

#### Heteronormative attitudes and beliefs

The Heteronormative Attitudes and Beliefs Scale [41] assessed participants’ level of endorsement of heteronormative standards. Specifically, participants were asked whether they believed that gender was a binary concept and whether gender was determined by sex, i.e., essentialist beliefs about sex and gender. Thereby, participants indicated their level of agreement to statements, such as, “Masculinity and femininity are determined by biological factors, such as genes and hormones, before birth” on a seven-point Likert scale (1 = totally disagree, 7 = totally agree). A second scale of the Heteronormative Attitudes and Beliefs Scale assessed participants’ level of agreement to sexual double standards concerning human sexual behavior, i.e., whether different standards and opportunities in regard to partnered sexual behavior should apply to women and men (e.g., “In intimate relationships, people should act only according to what is traditionally expected of their gender”). Essentialist beliefs about sex and gender were originally assessed with a reliability of α = 0.92 (eight items) and sexual double standards were originally assessed with a reliability of α = 0.78 (eight items) [41]. For the current study, the items were translated into German. Both scales had reliabilities higher than 0.8 (Table 1).

**Table 1:** *Descriptive Statistics and Group Comparisons*

Variable	All	Possible range	α	Gender		Subsample		Cluster		
				Men	Women	Students	Employees	1	2	3
Age	31.7 (12.3)			31.2 (12.6)	31.9 (12.1)	23.1 (3.1)	41.2 (11.6)^b^	32.6 (12.3)	31.6 (12.7)	28.7 (11.2)
Essentialist belief	2.8 (1.3)	1–7	0.89	3.4 (1.4)	2.6 (1.1)^a^	2.9 (1.4)	2.8 (1.1)	2.7 (1.1)^d, e^	3.5 (1.4)^c, e^	2.2 (0.8)^c, d^
Double standards	2.1 (0.9)	1–7	0.82	2.4 (0.9)	2.0 (0.8)^a^	2.0 (1.0)	2.2 (0.8)	2.1 (0.9)^d, e^	2.5 (0.9)^c, e^	1.6 (0.5)^c, d^
Knowledge	3.2 (1.9)	1–10	0.57	3.0 (1.9)	3.3 (1.9)	3.2 (2.0)	3.1 (1.9)	3.2 (1.9)^e^	2.8 (1.9)^e^	4.1 (2.1)^c, d^
Perceived affirmative action	1.5 (0.4)	1–5	0.63	1.6 (0.5)	1.5 (0.4)^a^	1.7 (0.4)	1.4 (0.4)^b^	1.5 (0.4)	1.6 (0.5)^c^	1.5 (0.4)
Importance of affirmative actions	3.0 (0.9)	1–5	0.90	2.6 (0.9)	3.2 (0.9)^a^	3.1 (0.9)	3.0 (0.9)	3.2 (0.9)^d, e^	2.5 (0.8)^c, e^	3.6 (0.8)^c, d^
Desire for certain affirmative actions	2.9 (1.1)	1–5	0.92	2.4 (1.0)	3.2 (1.0)^a^	3.0 (1.1)	2.9 (1.0)	3.1 (1.0)^d, e^	2.4 (1.0)^c, e^	3.5 (1.0)^c, d^

*Note*. ^a^Significant gender difference, *t*(224.0–418) = 5.2–6.6, *p* < .001, *d* = 0.5–0.7

^b^Significant differences between students and employees, *t*(222.8–417) = 6.4–21.3, *p* < .001, *d* = 0.6–2.2

^c^Significantly different from responses in Cluster 1, *F*(410–415, 2) = 6.4–30.4, *p* < .001, and post-hoc Bonferroni corrected *p* < .021

^d^Significantly different from responses in Cluster 2, *F*(410–415, 2) = 6.4–30.4, *p* < .001, and post-hoc Bonferroni corrected *p* < .001

^e^Significantly different from responses in Cluster 3, *F*(410–415, 2) = 6.4–30.4, *p* < .001, and post-hoc Bonferroni corrected *p* < .021

#### Knowledge test

The knowledge test was self-constructed and can be found in the supplemental materials (Table S1). Most of the questions are based on a (biomedical) review about intersex individuals [9], whereas some questions are specific to the Austrian law [15, 16]. Participants were asked whether ten statements about intersex persons were true or false or whether they did not know the answer. One example item is, “An intersex person needs medical help or intervention.” All correct responses were given one point and the points were added to form a total score. Participants with incorrect responses and participants who chose the response “I do not know” scored zero points on a respective knowledge question. The knowledge test had a reliability of 0.57.

#### Affirmative actions

A list of twelve affirmative actions (e.g., “When filling out forms persons with intersex identity can identify as intersex”; Supplementary Material, Table S2) was formulated based on previous literature and recommendations for implementing LGBT+, transgender and nonbinary affirmative spaces in graduate education [27–29]. Participants indicated on a five-point Likert scale (1 = never seen, 5 = always) how often they had noticed the listed affirmative actions at their medical university. The internal consistency of the sum score was above 0.6 (Table 1).

In a next step, participants received the same list of affirmative actions. This time they were asked to indicate how important they thought implementation of those affirmative actions was on a five-point Likert scale (1 = not at all; 5 = very important). This scale had an internal consistency of α = 0.90.

Finally, participants were asked to indicate on a list of ten affirmative actions (e.g., “I want more education opportunities (lectures, seminars) about gender-fair language;” Table S3) whether they wished that a particular affirmative actions would be implemented at their medical university. The list of affirmative actions was based on recommendations from the literature [27–29]. This scale had an internal consistency of α = 0.92.

### Statistical analysis

Descriptive statistics and *t*-tests were used to describe participants’ responses. A hierarchical latent cluster analysis was performed using an agglomerative approach, i.e., at first each case was treated as its own cluster until cases were sequentially merged and all cases formed a single cluster [42, 43]. Cases were defined by gender, sexual orientation, levels of essential belief about sex and gender, levels or endorsement of sexual double standards concerning sexual behavior, and knowledge about intersex variation. For the cluster analysis variables were *z*-transformed. For measuring distance the Squared Euclidean distance was calculated and Single Linkage was used for merging clusters [44]. Cluster solutions between two and four clusters were analyzed, whereas the final number of clusters was chosen based on the strongest increase in the heterogeneity coefficient [44]. The current study’s sample size seemed large enough for small to medium effects and weak to medium class separation conditions with a power of 0.8 [45]. Analyses of Variance (ANOVAs) with Bonferroni corrections for post-hoc analyses were performed to compare whether people grouped to different clusters reported different numbers of affirmative actions at the medical university, or whether they supported (i.e., perceived the importance of and wished for) affirmative actions to a different degree. The level of significance for the analyses was α = 0.05. All statistical analyses were performed with SPSS for Windows, version 26.0 (IBM Corp., Armonk, NY, USA).

## Results

### Participants

Responses from 420 participants were included. The sociodemographic characteristics of the sample are reported in Table 2. Nearly half of the sample were employees and the other half of the sample were students at the medical university. Of the employees half of the sample belonged to the academic staff and 40.0% of the sample belonged to the administrative staff. The mean age of employees was 41.2 (*SD* = 11.6, range: 20–70) years and the mean age of students was 23.1 (*SD* = 3.1, range: 18–38) years. Overwhelmingly women participated in the study. The majority of respondents identified themselves as being heterosexual and had Austrian nationality. Nearly half of the sample had finished school or vocational training, whereas the other half of the sample had a university degree.

**Table 2:** Sociodemographic description of the sample

Variable		All *N* (%)	Men *N* (%)	Women *N* (%)	Students *N* (%)	Employees *N* (%)
Gender						
	Man	141 (33.6)			84 (59.6)	57 (40.4)
	Woman	279 (66.4)			136 (48.7)	143 (51.3)
Subsample						
	Students	220 (52.4)	84 (38.2)	136 (61.8)		
	Employees	200 (47.6)	57 (28.5)	143 (71.5)		
Nationality						
	Austrian	337 (89.8)	127 (33.7)	250 (66.3)	182 (48.3)	195 (51.7)
	Other	41 (9.8)	14 (34.1)	27 (65.9)	37 (90.2)	4 (9.8)
Education						
	School or vocational training	228 (54.8)	81 (35.5)	147 (64.5)	185 (81.1)	43 (18.9)
	University	188 (45.2)	59 (31.4)	129 (68.6)	33 (17.6)	155 (82.4)
Roles of employees						
	Administrative staff	80 (19.0)	13 (22.8)	67 (46.9)		80 (40.0)
	Academic staff	101 (24.0)	39 (68.4)	62 (43.4)		101 (50.5)
	Third party founded staff	19 (4.5)	5 (8.8)	14 (9.8)		19 (9.5)
Sexual orientation						
	Heterosexual	356 (84.8)	124 (34.8)	232 (65.2)	179 (50.3)	177 (49.7)
	Sexual minority	64 (15.2)	17 (26.6)	47 (73.4)	41 (64.1)	23 (35.9)
Cluster						
	1	230 (55.0)	0 (0.0)	230 (100.0)	107 (46.5)	123 (53.5)
	2	124 (29.7)	124 (100.0)	0 (0.0)	72 (58.1)	52 (41.9)
	3	64 (15.3)	17 (26.6)	47 (73.4)	41 (64.1)	23 (35.9)

### Descriptive statistics

Descriptive results are reported in Table 1. Overall participants did *rather not* hold essentialist beliefs about sex and gender and participants did not agree with sexual double standards concerning human sexual behavior [41]. In the whole sample, the mean score of the knowledge test was three points. Thus, overall every participant responded one average to three knowledge test items correctly (Table 1).

On average participants noticed three affirmative actions at their medical university. The action most often noticed was the discussion of minority stress during meetings/lectures, the gender-neutral e-mail correspondence, and open identification of students/colleagues with an LGBTIQ + identity (Table S2). Participants also relatively often noticed that intersex identity could be indicated in official forms.

Overall, participants thought affirmative actions were moderately important (Table 1). Participants agreed that it was important that intersex could be documented on paperwork and in the patient register (Table S2). Furthermore, participants agreed that it was important that disadvantages experienced by minority groups be discussed during meetings/lectures and for LGBTIQ + students/colleagues to be able to openly identify with a minority identity (Table S2).

Most of the participants took a neutral standpoint toward the implementation of affirmative actions at their medical university (Table 1). The affirmative actions most often desired were more information by superiors/supervisors about gender-sensitive behavior, concrete guidelines for gender-fair language in diploma/master theses and more education (lectures, seminars) about sex and gender (Table S3).

### Groups with different beliefs and wishes for affirmative action

The largest change in the heterogeneity coefficient was seen after the fusion of the four-cluster solution to a three-cluster solution (0.8; the heterogeneity coefficient change between the five- and four-cluster solutions was 0.3 and the heterogeneity coefficient change between the three- and two-cluster solutions was 0.0). In the three-cluster solution around half of the sample was grouped in the first cluster (Table 2). Around 30% of the participants were grouped in Cluster 2 and the remainder of the sample constituted Cluster 3 (Table 2). Thereby, all participants grouped in Cluster 1 were heterosexual cisgender women, all participants grouped in Cluster 2 were heterosexual cisgender men, and all participants grouped in Cluster 3 were sexual minority persons^1^.

Cluster 3 was further characterized as having participants with the lowest levels of essential beliefs about sex and gender and the lowest levels of endorsement of sexual double standards (Table 1). Cluster 2 was characterized as having participants with the highest levels of essential beliefs and the highest levels of endorsement of sexual double standards. Participants in Cluster 3 had the highest score on the knowledge test (Table 1).

There was no difference in the number of affirmative actions perceived at the medical university between clusters. The wish for more affirmative actions and the perceived importance of such actions was greatest among participants in Cluster 3 and poorest among participants in Cluster 2 (Table 1).

## Discussion

Even though intersex variation has been officially recognized by Austrian law as a “third sex” since 2018 [15, 16], the current study reveals that only few of the affirmative actions recommended with a view to ensuring inclusivity for intersex persons at universities [27, 28, 33] have been implemented at an Austrian medical university. Furthermore, students and employees have poor knowledge about intersex variation. However, students and employees did not hold strong heteronormative standards and moderately supported the implementation of affirmative actions.

The affirmative action most often noticed at the medical university was that university forms included the possibility to choose the response option intersex. Thus, intersex persons are no longer forced to identify with the sex categories female or male when filling out university forms and documents, but can register their sex as “inter.” [15, 16] Additionally, gender-neutral language has been implemented in e-mail correspondence. This change seems to have been accompanied by the discussion during meetings or lectures about disadvantages experienced by minority groups.

Even though those were the affirmative actions most often encountered at the medical university, only around half of the sample witnessed such changes and of those who saw affirmative actions implemented, most indicated having seen them “now and then.” Thus, the implementation of affirmative actions seems to be inconsistent throughout the medical university. However, for the increase in societal safety a more consistent indication of the medical university as a safe and inclusive space for intersex persons would be beneficial [24, 33]. This conclusion that the university provides inconsistent messages about being a safe space for sexual- and gender-minority groups is reflected in the finding that only half of students and employees have witnessed another student or employee of the university openly identifying with a sexual- or gender-minority identity.

One relatively easy way to implement an affirmative action for increasing intersex persons’ (or gender and sexual minorities’) sense of safety would be the use of symbols that transport inclusivity of sexual- and gender-minority persons. Such symbols can be placed in halls, waiting rooms, or lecture halls [27–29]. Increasing the number of and creating inclusive workplaces for intersex persons (and other sexual and gender minorities) can increase students’ and employees’ productivity and health [33, 46]. The current study found that the medical university has not (yet) used such symbols to indicate inclusivity for intersex or other sexual- and gender-minority persons.

Even though students and employees have perceived an increase in the number of discussions in meetings or lectures concerning discrimination experienced by minority groups, those discussions might not be specifically focusing on intersex variation. This conclusion was supported by the poor results on the current study’s knowledge test about intersex variation of most students and employees. Those findings are in line with results from other studies that reported a poor general understanding of intersex variation in the public [34, 47] or among healthcare professionals [48, 49]. Furthermore, many people adopt a medical perspective on intersex variation and perceive intersex variation as a medical condition needing medical intervention [34]. Such a perspective can have profound negative medical and psycho-social consequences for the intersex persons [5–8].

In order to increase students’ and employees’ understanding of intersex persons’ difficulties and challenges when not being able to conform to cis-heteronormativity expectations [7, 20] students and employees also need to understand the socio-psychological consequences of cis-heteronormativity on sexual- and gender-minority lives. Thereby, medical university staff and students need to understand power relations and challenge the (unjustified) privilege enjoyed by certain people who meet society’s expectations of cis-heteronormativity [36, 46]. In the current study especially those meeting expectations of cis-heteronormativity and those who might acquire privileges in gender-biased organizations (i.e., heterosexual cisgender men) [50–53] reported the least amount of support for affirming actions for intersex persons and the highest levels of endorsement of heteronormative standards.

Currently, medical education in German-language countries lacks educational or training programs specifically devoted to sexual- and gender-minority health [54–56]. Such programs need to be implemented and include sexual and gender-minorities’ lived perspectives in order to exemplify how pressures to comply with cis-heteronormativity can interfere with and be detrimental for sexual and gender minorities’ lives, health, and wellbeing [57].

As was the case in other studies about this topic [49], students and employees at the studied medical university would overwhelmingly welcome more education (lectures, seminars) about sexuality and gender. In the current study supervisors or persons in leading positions might need additional or focused training, because many participants expected their supervisors or superiors to be knowledgeable about inclusivity and gender-sensitive behavior. This request highlights the important role of supervisors in creating affirmative and inclusive workspaces [36]. Additionally, participants agreed that written guidelines and policies should be implemented [27–29].

One affirmative action, namely equal access to restrooms [27–29, 31], was not implemented at the studied medical university and only half of the participants wanted all gender restrooms (i.e., restrooms that are not segregated by gender). However, all gender restroom are one important way to challenge sex and gender binarism [32]. Persons who do not meet cis-heteronormative expectations might perceive strictly gendered spaces, such as gender-segregated restrooms as challenging and as a source of minority stress [58]. Maybe an increase in understanding how gendered spaces reinforce sex and gender binarism [32], thus reproduce and exemplify cis-heteronormativity, might convince more students and employees to advocate equal access to restrooms for intersex persons and other persons who do not comply with gender binarism.

Participants reported that for certain positions (e.g., tenure track, professorship) or functions (e.g., board membership, work council) no quotas were in place for sexual and gender minorities. Similar to the opinion about gender quotas [59, 60], the suggestions on implementing quotas for sexual and gender minorities met with some opposition from participants. More than half of the participants did not support the idea of implementing quotas for sexual- and gender-minority individuals when considering candidates for certain positions or functions [61]. This opposition might be routed in a zero-sum perspective, i.e., the belief that gains made in one group (e.g., currently marginalized group) translate to an equivalent loss for another group (e.g., currently privileged) [61].

Even though many participants reported that some paperwork (i.e., forms) at the medical university included the option intersex, such changes have not been implemented in the patient register. The overwhelming majority of students and employees reported not having the possibility to record intersex in the patient register. The current study’s results are in line with previous research results [49]. Therefore, the adaptation of the patient register for options beyond binary categories of sex and gender is called for. The majority of the medical university’s students and employees agreed that a more inclusive patient register is important.

### Implications

One recommendation or first step would be to implement more education (lectures, seminars) about sexuality and gender, specifically about intersex variation. The majority of the current study’s respondents would welcome such educational programs. It is recommended that such educational programs not focus solely on biomedical aspects of intersex variation but help participants understand the power relations, namely that strict endorsement of cis-heteronormativity gives certain people who meet those society’s expectations (unjustified) privileges over other people who do not meet those standards [36, 46, 57]. Maybe after understanding such power relations and resulting forms of discrimination that sexual and gender minorities experience the support for currently moderately supported affirmative actions will increase.

Future longitudinal studies are needed to evaluate how suggested educational programs influence students’ and employees’ opinions about affirmative actions for intersex persons. It would be of interest to know whether such educational programs can reduce the currently found differences in support for affirmative actions between men and women or between heterosexual and sexual minority persons. Other studies could focus on finding ways to optimally support supervisors or persons in leading positions to implement affirmative actions, because many participants in the current study expected their superiors and supervisors to be knowledgeable and to take steps to create more inclusive work environments.

### Limitations

When interpreting the current study’s results some key limitations need to be considered. The generalizability of the current study is limited because the study is based on findings from a single university and on a relatively small number of students and employees. Another consequence of including only one organization and having a small number of participants is the weak representation of gender-diverse participants in the study. The number of participants with nonbinary, transgender or other gender identity was too small for those responses to be included in the analysis. Additionally, reporting responses based on such a small subgroup might have given rise to concerns about the possibility to preserve those participants’ anonymity (especially when reporting other sociodemographic characteristics of the small subgroup). Additionally, the perspective of intersex persons is missing in the current study. Future multicenter studies in Austria are needed to include the perspective of gender-diverse university members.

Another limitation is the self-constructed knowledge test. The knowledge test was constructed based on scientific literature about intersex variability [9] and some questions were specific to Austrian law [15, 16]. Additional steps, such as pre-testing the knowledge test with a group of experts, pre-assessing and evaluating psychometric properties of single items, or hypothesizing and testing specific factor structures of the scale, could have improved the knowledge test’s quality. Other knowledge tests in the literature assessing knowledge about sexual and gender minorities’ health [48, 49] have only few items specific to intersex variability and therefore were not used in the current study. The relatively low alpha coefficient of the knowledge test in the current study might indicate that not all items assess the same construct (i.e., knowing about Austrian law concerning intersex variation might be independent of knowing biomedical facts about intersex variation).

Similarly, low internal consistencies in the score representing how many affirmative actions are implemented at the medical university might have resulted because the implementation of a particular affirmative actions is independent of the implementation of a different affirmative action (thus, certain item scores might not strongly correlate with the sum score). For this reason, the exact responses to each affirmative action are reported in the Supplementary Material (Table S2 and Table S3) and each affirmative action is discussed separately in the Discussion.

As is the case with most questionnaire studies, the current study is based on self-reports and socially desirable responding of participants may have biased results. Finally, the cross-sectional study design precludes any conclusions of causality.

## Conclusion

After the Austrian law has officially recognized intersex variation as a “third sex” and thus, contributed a significant step forward in reducing discrimination of intersex persons, universities should implement affirmative actions, i.e., proactive practices to avert discrimination. At the studied Austrian medical university, more and most notably more consistent affirmative actions for the inclusion of intersex people are needed. The current study has revealed that only few affirmative actions that challenge sex and gender binarism or that create inclusive and safe workplaces for intersex persons have been implemented at the medical university.

### Electronic supplementary material

Below is the link to the electronic supplementary material.

**Supplementary Material 1**: Table S1: Self-Constructed Knowledge Test Items.

**Supplementary Material 2**: Table S2: Exact Wording of Items for Assessing Perceived Affirmative Actions.

**Supplementary Material 3**: Table S3 Exact Wording of Items for Assessing Desire for Certain Actions to be Implemented.

## Data Availability

The datasets used and/or analyzed during the current study are available from the corresponding author on reasonable request.

## Footnotes

1 The four-cluster solution included the same Cluster 1 (cisgender women; *n* = 230; 55.0%) and Cluster 2 (cisgender women; *n* = 124; 29.7%) as the three-cluster solution. Sexual minority persons were grouped in Cluster 3 (sexual minority cisgender women; *n* = 47; 11.2%) and Cluster 4 (sexual minority cisgender men; *n* = 17; 4.1%) in the four-cluster solution.

## Declarations

### Previous presentations

No previous presentations.
