# Supplementary material for: Support for affirmative actions to increase inclusivity of intersex* persons at an Austrian medical university
Source: BMC Med Educ. 2023 Nov 3;23:825. doi: 10.1186/s12909-023-04830-z (PMC10623750; doi:10.1186/s12909-023-04830-z)
Supplement: Supplementary file 1 — Supplementary Material 1: Table S1: Self-Constructed Knowledge Test Items. [file 12909_2023_4830_MOESM1_ESM.docx]

**Table S1**

*Self-Constructed Knowledge Test Items*

| **Question German** | **Question English** | **Correct response** | **Participants who responded correctly (%)** | **Comment** |
| --- | --- | --- | --- | --- |
| Als Personen mit dem Geschlecht divers werden jene Personen verstanden, die sich weder als Mann oder Frau fühlen bzw. identifizieren. | Intersex persons are defined as persons who neither feel nor identify as woman or man. | false | 11.2 | This description better fits the identity nonbinary. Further, this definition does not take physical traits into account, which are key determinants of intersex variation according to Austrian law.^1, 2^ |
| Ein anderer Begriff für das Geschlecht divers ist Intersex. | Another term for “third sex” is intersex. | true | 29.0 | According to Austrian law the term “diverse” is used interchangeably for intersex variation.^1, 2^ |
| Bei Personen mit dem Geschlecht divers stimmen das „soziale“ und „biologische“ Geschlecht nicht überein. | In intersex persons the sex assigned at birth and the gender do not match. | false | 25.3 | This description better fits the identity transgender. Further, this definition does not take physical traits into account, which are key determinants of intersex variation according to Austrian law.^1, 2^ |
| Eine Person mit dem Geschlecht divers benötigt medizinische Hilfe bzw. Intervention. | An intersex person needs medical help or intervention. | false | 80.7 | Even though some intersex variations are accompanied by congenital medical problems, most intersex persons do not require medical care.^3^ |
| Ein anderer Begriff für das Geschlecht divers ist „Varianten der Geschlechtsentwicklung“. | Another term for “third sex” is variation in sex development. | true | 30.5 | According to Austrian law the term “diverse” is used interchangeably for variation in sex development.^1, 2^ |
| Personen mit zwei X-Chromosomen, die einen Penis haben, gehören dem Geschlecht divers an. | Persons with two X chromosomes and a penis are intersex persons. | true | 24.8 | The example describes a person with 46, XX or 47, XXY variation in sex development.^3^ |
| Personen mit dem Geschlecht divers wünschen ein anderes Geschlecht, als jenes, das ihnen bei der Geburt zugewiesen wurde. | Intersex persons want to be identified as having a gender different than the sex assigned to them at birth. | false | 44.0 | This description better fits the identity transgender. Further, this definition does not take physical traits into account, which are key determinants of intersex variation according to Austrian law.^1, 2^ |
| Personen mit dem Geschlecht divers haben eine atypische Entwicklung des biologischen (chromosomalen, anatomischen und/oder hormonellen) Geschlechts. | Intersex persons have atypical development of their biological sex characteristics (chromosomal, gonadal, and/or anatomical sex). | true | 24.1 | This is the definition for intersex variation used by some authors.^4^ |
| Um das Geschlecht divers im Zentralen Personenstandsregister und in den personenstandsrechtlichen Urkunden eintragen zu können, benötigt eine Person ein medizinisches Gutachten. | In order to be registered as intersex in the official (the country’s) personal register the person needs a medical examination and approval. | true | 26.2 | According to Austrian law intersex persons can register as intersex in the official (the country’s) personal register only after undergoing a medical examination and receiving confirmation of physical characteristics.^1, 2^ |
| Um das Geschlecht divers im Zentralen Personenstandsregister und in den personenstandsrechtlichen Urkunden eintragen zu können, benötigt eine Person ein psychologisches Gutachten. | In order to be registered as intersex in the official (country’s) personal register the person needs a psychological examination and approval. | false | 21.9 | According to Austrian law no psychological examination or approval is needed to enter intersex in the official (country’s) personal register.^1, 2^ |

## References

1. Bierlein B. Verfahren zur Prüfung der Verfassungsmäßigkeit des § 2 Abs. 2 Z 3 des Bundesgesetzes über die Regelung des Personenstandswesens (Personenstandsgesetz 2013 – PStG 2013), BGBl. I 16/2013. *G 77/2018-9*: Verfassungsgerichthof; 2018.

2. Hudsky D. Verwaltungsangelegenheiten - Sonstige; Personenstandswesen Erkenntnis des VfGH vom 15. Juni 2018, G 77/2017-9, zu § 2 Abs. 2 Z 3 PStG 2013 - Umsetzung zu Varianten der Geschlechtsentwicklung ("3. Geschlecht"). In: Inneres B, ed.: Bundesministerium Inneres; 2018.

3. Cools M, Nordenström A, Robeva R, et al. Caring for individuals with a difference of sex development (DSD): A consensus statement. *Nat Rev Endocrinol*. 2018;14: 415-29.

4. Lee PA, Nordenström A, Houk CP, et al. Global disorders of sex development update since 2006: Perceptions, approach and care. *Horm Res Paediatr*. 2016;85: 158-80.
