# Supplementary material for: Support for affirmative actions to increase inclusivity of intersex* persons at an Austrian medical university
Source: BMC Med Educ. 2023 Nov 3;23:825. doi: 10.1186/s12909-023-04830-z (PMC10623750; doi:10.1186/s12909-023-04830-z)
Supplement: Supplementary file 2 — Supplementary Material 2: Table S2: Exact Wording of Items for Assessing Perceived Affirmative Actions. [file 12909_2023_4830_MOESM2_ESM.docx]

**Table S2**

*Exact Wording of Items for Assessing Perceived Affirmative Actions and Perceived Importance of Certain Actions*

| Affirmative action | Never seen/Not at all *n* (%) | Seen once/Rather not *n* (%) | Every now and then/moderate *n* (%) | Often seen/important *n* (%) | Always seen/very important *n* (%) |
| --- | --- | --- | --- | --- | --- |
| At my university there are specified restrooms for intersex persons. |  |  |  |  |  |
| Affirmative action perceived | 401 (95.5) | 7 (1.7) | 10 (2.4) | 1 (.2) | 1 (.2) |
| Importance of affirmative action | 90 (23.0) | 80 (20.4) | 84 (21.4) | 103 (26.3) | 35 (8.9) |
| **When filling out forms persons with intersex identity can identify as intersex.** |  |  |  |  |  |
| Affirmative action perceived | 241 (57.4) | 7 (1.7) | 69 (16.4) | 67 (16.0) | 36 (8.6) |
| Importance of affirmative action | 24 (5.9) | 17 (4.2) | 43 (10.6) | 121 (30.0) | 199 (49.3) |
| **It is possible to indicate intersex identity in the patient register.** |  |  |  |  |  |
| Affirmative action perceived | 345 (82.1) | 5 (1.2) | 30 (7.1) | 27(6.4) | 13 (3.1) |
| Importance of affirmative action | 25 (6.2) | 16 (4.0) | 32 (8.0) | 114 (28.4) | 214 (53.4) |
| My supervisor/superior asks me about my preferred pronouns. |  |  |  |  |  |
| Affirmative action perceived | 411 (97.9) | 1 (0.2) | 5 (1.2) | 1 (0.2) | 2(0.5) |
| Importance of affirmative action | 105 (26.1) | 67 (16.7) | 101 (25.1) | 85 (21.1) | 44 (10.9) |
| In e-mail correspondence gender-neutral expressions are used to address the addressee. |  |  |  |  |  |
| Affirmative action perceived | 224 (53.3) | 15 (3.6) | 77 (18.3) | 53 (12.6) | 50 (11.9) |
| Importance of affirmative action | 82 (20.1) | 47 (11.5) | 82 (20.1) | 101 (24.8) | 95 (23.3) |
| My colleagues asks me about my preferred pronouns. |  |  |  |  |  |
| Affirmative action perceived | 405 (96.4) | 9 (2.1) | 4 (1.0) | 2 (0.5) | 0 (0.0) |
| Importance of affirmative action | 162 (39.9) | 86 (21.2) | 80 (19.7) | 49 (12.1) | 29 (7.1) |
| Symbols that transport inclusivity are seen in halls/waiting rooms/lecture halls. |  |  |  |  |  |
| Affirmative action perceived | 360 (85.7) | 13 (3.1) | 35 (8.3) | 10 (2.4) | 2 (0.5) |
| Importance of affirmative action | 92 (23.2) | 43 (10.8) | 72 (18.1) | 126 (31.7) | 64 (16.1) |
| **Disadvantages experienced by minority groups are discussed in meetings/lectures.** |  |  |  |  |  |
| Affirmative action perceived | 196 (46.7) | 30 (7.1) | 134 (31.9) | 48 (11.4) | 12 (2.9) |
| Importance of affirmative action | 29 (7.2) | 21 (5.2) | 57 (14.1) | 125 (30.9) | 172 (42.6) |
| Students/Colleagues wear symbols (scarf, shoes, stickers) that identify them as having an LGBTIQ+ identity. |  |  |  |  |  |
| Affirmative action perceived | 291 (69.3) | 33 (7.9) | 79 (18.8) | 17 (4.0) | 0 (0.0) |
| Importance of affirmative action | 247 (63.2) | 73 (18.7) | 45 (11.5) | 18 (4.6) | 8 (2.0) |
| **Students/Colleagues feel comfortable with openly identifying with an LGBTIQ+ identity.** |  |  |  |  |  |
| Affirmative action perceived | 220 (52.4) | 40 (9.5) | 116 (27.6) | 38 (9.0) | 6 (1.4) |
| Importance of affirmative action | 92 (23.5) | 23 (5.9) | 31 (7.9) | 54 (13.8) | 192 (49.0) |
| Quotas for certain positions (tenure track, professorship) for people who identify as LGBTIQ+ are in place. |  |  |  |  |  |
| Affirmative action perceived | 407 (96.9) | 6 (1.4) | 6 (1.4) | 0 (0.0) | 1 (0.2) |
| Importance of affirmative action | 143 (38.2) | 71 (19.0) | 76 (20.3) | 60 (16.0) | 24 (6.4) |
| Quotas for certain functions (board membership, work council) for people who identify as LGBTIQ+ are in place. |  |  |  |  |  |
| Affirmative action perceived | 409 (97.4) | 7 (1.7) | 3 (0.7) | 0 (0.0) | 1 (0.2) |
| Importance of affirmative action | 140 (36.7) | 66 (17.3) | 76 (19.9) | 72 (18.9) | 27 (7.1) |

*Note*. Bold implementations are perceived as moderate to very important by the majority of the sample.
