# Supplementary material for: Support for affirmative actions to increase inclusivity of intersex* persons at an Austrian medical university
Source: BMC Med Educ. 2023 Nov 3;23:825. doi: 10.1186/s12909-023-04830-z (PMC10623750; doi:10.1186/s12909-023-04830-z)
Supplement: Supplementary file 3 — Supplementary Material 3: Table S3 Exact Wording of Items for Assessing Desire for Certain Actions to be Implemented. [file 12909_2023_4830_MOESM3_ESM.docx]

**Table S3**

*Exact Wording of Items for Assessing the Desire for Certain Actions to be Implemented*

| Affirmative action | Totally disagree *n* (%) | Rather disagree *n* (%) | Moderately agree *n* (%) | Rather agree *n* (%) | Totally agree *n* (%) |
| --- | --- | --- | --- | --- | --- |
| **I want my superiors/supervisors to be better informed about gender-sensitive behavior.** | 61 (14.8) | 49 (11.9) | 82 (19.9) | 148 (35.9) | 72 (17.5) |
| **I want more education opportunities (lectures, seminars) about gender.** | 58 (14.3) | 55 (13.6) | 84 (20.7) | 131 (32.3) | 77 (19.0) |
| **I want more education opportunities (lectures, seminars) about gender-fair language.** | 73 (17.9) | 58 (14.2) | 77 (18.9) | 112 (27.5) | 88 (21.6) |
| I want quotas that reflect intersex identity. | 140 (37.0) | 92 (24.3) | 76 (20.1) | 52 (13.8) | 18 (4.8) |
| **I want affirmative action programs for people with intersex identity.** | 90 (23.4) | 54 (14.0) | 83 (21.6) | 111 (28.8) | 47 (12.2) |
| I want all gender restrooms (restrooms that are not segregated by gender). | 125 (31.8) | 74 (18.8) | 58 (14.8) | 67 (17.0) | 69 (17.6) |
| I want restrooms for people with intersex identity. | 132 (34.6) | 73 (19.2) | 80 (21.0) | 72 (18.9) | 24 (6.3) |
| **I want the form of address “Ladies and Gentlemen” to be replaced with a gender-neutral form of address.** | 98 (24.7) | 51 (12.9) | 80 (20.2) | 98 (24.7) | 69 (17.4) |
| **I want the “capital I” used in gender-fair forms of gendered German words to be replaced with an asterisk (*) or underline (_).** | 97 (29.6) | 43 (13.1) | 56 (17.1) | 74 (22.6) | 58 (17.7) |
| **I want the university to publish guidelines for gender-fair language in diploma/master theses.** | 78 (19.5) | 37 (9.3) | 65 (16.3) | 112 (28.0) | 108 (27.0) |

*Note*. Bold implementations are moderately to totally desired by most participants.
